# Norethindrone is superior to combined oral contraceptive pills in short-term delay of menses and onset of breakthrough bleeding: a randomized trial

**DOI:** 10.1186/s12905-019-0766-6

**Published:** 2019-05-28

**Authors:** Joshua Dean, Katherine J. Kramer, Fauzia Akbary, Shaunte Wade, Maik Hüttemann, Jay M. Berman, Maurice-Andre Recanati

**Affiliations:** 1 School of Medicine, Wayne State University, Detroit, MI 48201 USA; 2 Department of Obstetrics and Gynecology, St. Vincent’s Catholic Medical Centers, New York, NY 10011 USA; 3 Wayne State University School of Medicine, Detroit, MI 48201 USA; 4 Center for Molecular Medicine and Genetics, Wayne State University School of Medicine, Detroit, MI 48201 USA; 5 Department of Obstetrics and Gynecology, Wayne State University, Detroit, MI 48201 USA; 6 NIH-Women’s Reproductive Health Research (WRHR) Scholar, Department of Obstetrics and Gynecology, Wayne State University, Detroit, MI 48201 USA

**Keywords:** Norethindrone, Menstruation delay, Randomized controlled study, Vaginal bleeding

## Abstract

**Background:**

To determine whether oral norethindrone acetate is superior to combined oral contraceptives (OCP) in delaying menstruation and preventing breakthrough bleeding when started late in the cycle.

**Methods:**

This article comprises of a case control study followed by a pilot randomized controlled study. In the first study, four women who presented late in their cycle and desired avoiding vaginal bleeding within 10 days before a wedding were started on norethindrone 5 mg three times daily and compared to age matched controls started on OCPs. Subsequently, a randomized controlled pilot study (*n* = 50) comparing OCPs to norethindrone for the retiming of menses was conducted. Percentage of women reporting spotting were compared with level of statistical significance set at *p* < 0.05.

**Results:**

Of the norethindrone treated group, only 2 women (8%) reported spotting compared with 10 women (43%) in the control group (*p* < 0.01). Norethindrone recipients experienced significant weight gain, which resolved after cessation of therapy and had heavier withdrawal bleed (*p* < 0.04) when compared to controls. Patient satisfaction was significantly higher in the norethindrone group, with 80% willing to choose this method again. Time to conceive was significantly shorter in the norethindrone group (*p* < 0.03).

**Conclusions:**

Norethindrone, begun on or before cycle day 12, is superior for women who desire to avoid breakthrough bleeding and maintain fertility when compared to OCPs. It is an ideal approach in patients presenting late in their cycle and who desire delaying menses as well as in circumstances when even minute amounts of breakthrough bleeding cannot be tolerated.

**Trial registration:**

Clinicaltrials.gov NCT03594604, July 2018. Retrospectively registered.

## Background

The culturally competent OBGYN should consider social, cultural and religious aspects when counselling patients. Menstruation can interfere with activities such as camping and sports, thus women choose to postpone their periods for practical reasons. For those not on hormonal birth control, the timing between the onset of the intervention and the planned event, as well as the tolerance for some amount of spotting early-on, must be contemplated.

Under Islamic law, menstruating Muslim women are prevented from participating in religious life, engaging in sexual intercourse [1], and from participating in the annual Hajj to Mecca. In a survey of Muslim Hajj pilgrims, fear of menstruation produced feelings of anxiety and a sense of rejection [2]. The consensus among Islamic scholars is that menstrual suppression is appropriate allowing women to fully engage in religious activities [3]. Despite religious approval, medical intervention is attainable only by wealthy and educated members of Islamic society as access is limited by cost [3].

Orthodox Jewish women closely follow Torah (Old Testament) laws. The commandment to be “fruitful and multiply” is balanced by laws of “family purity”, stating that a menstruating woman is in Niddah (ritually impure) from the beginning of menses until seven days after she is no longer shedding uterine blood. During this time, and until she immerses herself in a ritual bath (mikva) on the seventh day, she may not have physical contact with her husband. Orthodox women with prolonged menses, abnormal uterine bleeding or a short follicular phase might be unable to perform mikva, rendering them religiously infertile [4].

Since spotting renders women unclean in many traditions, gynecologists must carefully select treatment methods. This is especially important in the case of Jewish brides seeking to consummate their marriage on their wedding night. In many cases, these women present relatively late for medical intervention, sometimes just weeks before their wedding. Many also desire to start a family shortly after marriage, thus posing a unique challenge.

Many brides choose to initiate OCPs three months prior to the wedding and time the period appropriately. However, this method has a significant incidence of failure (especially in the first few months of treatment) due to breakthrough bleeding, thus rendering a woman Niddah. To prevent this, some women choose to self-administer increased dosage despite the risk of thromboembolism [5]. Depot-medroxyprogesterone acetate (DMPA) injections as well as hormone releasing intrauterine devices (IUDs) may render patients amenorrheic but often causes unpredictable spotting early in the course of treatment. DMPA may also cause a delay in the return to fertility once treatment is stopped.

Norethindrone, an orally active progestational agent, has been shown to be effective in preventing breakthrough (unscheduled) bleeding and has a mild side-effect profile, making it a desirable alternative [6]. We report on a case study of four women who were seen at St. Vincent’s Hospital and which prompted a pilot randomized control study comparing norethindrone with OCPs in preventing vaginal bleeding.

## Methods

Under IRB approval, women presenting to the Resident’s Obstetrics and Gynecology clinic at St. Vincent’s Medical Center in Manhattan and who desired delaying menstruation for any reason were included in this study. This included Orthodox Jewish brides (*n* = 4), as part of the case-control study, and a randomized-controlled study consisting of (*n* = 50) patients. A similar number of women served as controls. Number of women reporting spotting in each group were compared using Chi-square (significance set at *p* < 0.05).

### Case control study consisting of orthodox Jewish brides (n = 4)

Treatment with 5 mg norethindrone acetate (Teva Pharmaceuticals) three times daily was begun on or before day 12 of the menstrual cycle before the wedding. Norethindrone was begun at least 3 weeks (range 3–4 weeks) before the wedding day and continued until the marriage was consummated. Treatment duration never exceeded 35 days. Patients who missed a dose were told to take the medication as soon as they remembered to. The mean age was 22 (range 18–26) and nulligravida. A control group consisted of 4 married women from the same ethnic background and age group who desired contraception and were started on OCPs the day of their visit and instructed to use a back-up method for 7 days. A questionnaire at follow-up determined presence of spotting and side-effects. Researchers were blinded to the treatment group.

### Randomized control study of women who desired delaying menstruation (*n* = 23)

**Fig. 1:**
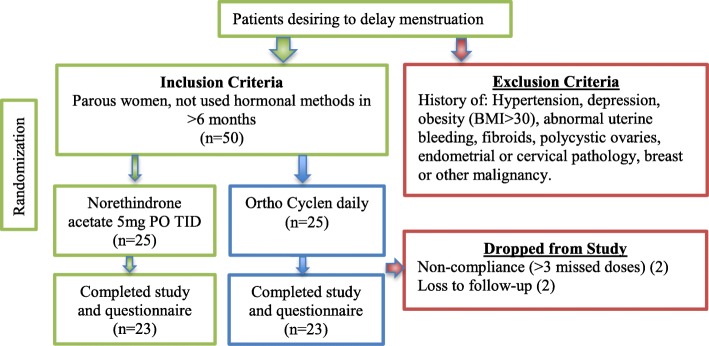
Randomized control Study design and patient inclusion. Two patients were removed from the study for non-compliance

**Fig. 2:**
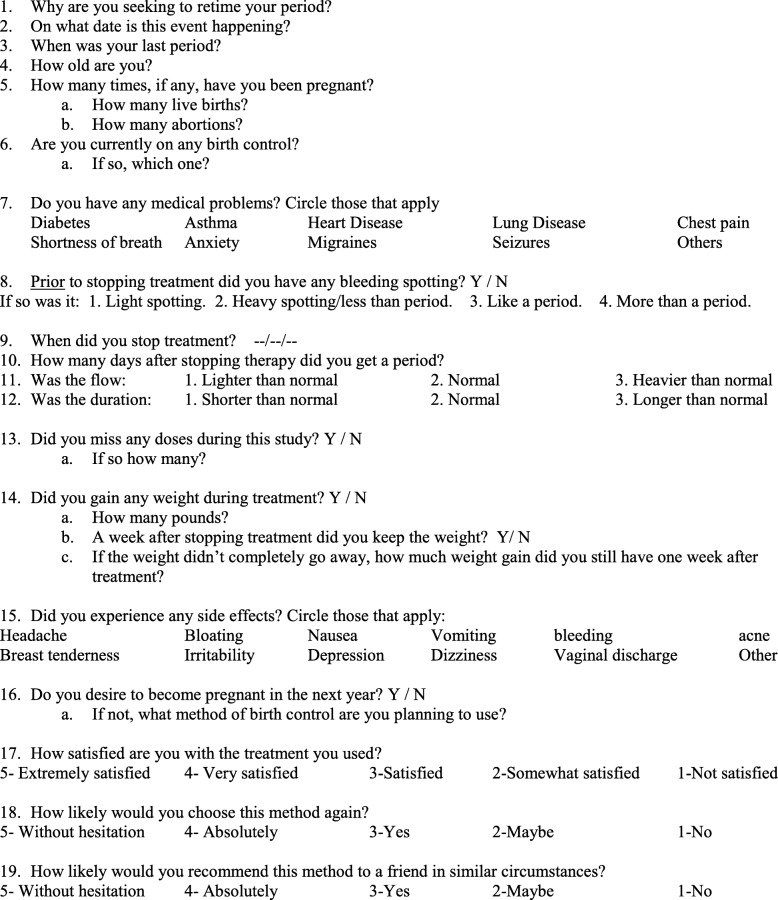
Questionnaire given to patients at the end of the study

A total of fifty women desiring to delay menstruation for any reason participated. Patients gave informed consent and were randomized at the time of enrolment by clinicians, using sealed envelopes, to receive either daily Ortho Cyclen birth control pills (with 2 daily placebos) or norethindrone three times daily. The tablets were dissimilar but not specifically branded. To avoid recall bias, the patients were given a copy of the survey which would be collected at the second visit, and asked to fill it out in real-time and to monitor their weight closely over the treatment and for at least 2 weeks thereafter. The average daily weight during treatment, as well as post-treatment, was compared to the pretreatment weight. Four patients were dropped from the study because of loss to follow-up or missing > 3 doses. Norethindrone (*n* = 23), prescribed similarly to the former group, was begun from 3 to 6 weeks before the upcoming event (trip/sporting event) and continued until menstruating was no longer inconvenient (treatment time: 28–45 days). The group consisted of women aged 18–33 (mean 28). The control group (n = 23) were started on same day OCPs (Fig. 1). Women with a history of hypertension, depression, obesity (BMI > 30), abnormal uterine bleeding, fibroids, polycystic ovarian syndrome, endometrial or cervical pathology or breast malignancy were excluded from participation. Parous women and women who had not used hormonal methods of contraception for > 6 months were included. Women completing the study were asked for their completed questionnaire (Fig. 2) at their second visit querying bleeding patterns, side-effects, satisfaction, weight gain and future reproductive goals. Investigators, who were not part of the recruitment team and had never met the patients, were blinded to treatment group and only received the completed surveys to analyze. A follow up phone call was conducted 6 months after the conclusion of the study by the clinicians in order to determine if the subject conceived. The timing of conception, based on stated due date, was provided to the blinded investigators and compared to treatment stop date.

## Results

**Fig. 3:**
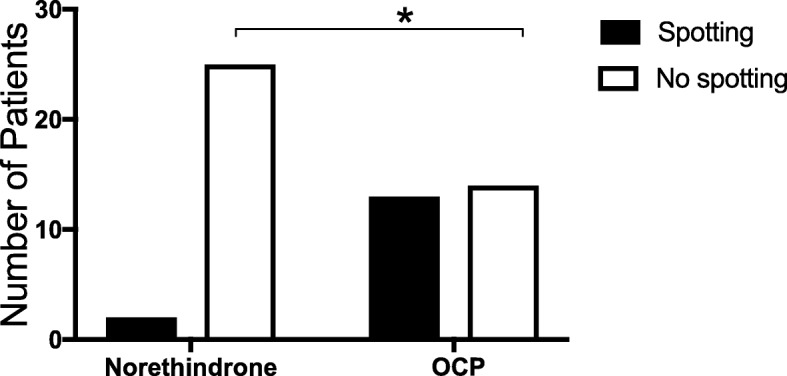
Incidence of Vaginal Bleeding or Spotting on Therapy Comparison of patients treated with norethindrone 5 mg three times daily versus controls treated with combined oral contraceptive pills. Aggregate data from both studies shown. (*n* = 27, *p* < 0.002)

Combined data are shown in Fig. 3 and subgroups are discussed below:

### Case control study consisting of orthodox Jewish brides

All four women who received norethindrone were free from bleeding during their wedding night and for the seven days prior. Patients were satisfied with this primary outcome and would recommend this method to a friend. Menstruation typically began on day 3 [2–4] after discontinuation and was slightly heavier than normal. Two women complained of fluid retention which resolved shortly after stopping norethindrone.

We compared this group with age matched controls who were treated with OCPs and, in this group, two women (out of 4) complained of spotting within the first few weeks of initiating therapy. Despite reassurance, one woman chose to switch to another birth control pill at the 3-month method check due to ongoing spotting. Because statistical power was too low to show significance, a randomized control study with larger sample size was undertaken.

### Randomized control study of women who desired delaying menstruation

Patients were randomly assigned into each arm of the study and groups were statistically similar in age, race, parity, cycle day of treatment initiation, days on therapy and desire to conceive within a year. Women treated with norethindrone denied any spotting while on therapy, with the exception of one woman who reported an incident of post-coital bleeding and one patient erroneously enrolled after cycle day 12 (Table 1). Ten women from the controls reported spotting while regularly taking OCPs (*p* < 0.007). Patient satisfaction with norethindrone was significantly higher than controls and 80% of patients would choose to use this method again, however weight gain was cited as a complaint by four (of 23) women. Although the norethindrone group had significantly more weight gain than controls, this resolved after cessation of treatment. While there was no statistical difference in number of patients reporting side-effects, the study suggests that norethindrone users experienced fewer issues. Withdrawal bleed was similar in timing, and duration between groups. Controls were significantly more likely to report bleeding lighter or equal to a menstrual period than the norethindrone group. Eleven women from the norethindrone group, and a similar number of women in the control group, chose to subsequently transition to hormonal or long-acting reversible contraceptive (LARCs) methods of birth control. Return to fertility was statistically significantly faster in the norethindrone group. No pregnancies or adverse effects were reported in any group during the study period.

**Table 1:** Patient Demographics and summary of results of randomized trial

Estimated	Norethindrone	Control Group (OCP)	p
N	23	23	
Age	27.7 (18–33) (0.883)	28.0 (18–33) (.916)	0.865
Race	White (26%), Hispanic (26%)	White (30%), Hispanic (18%)	ns
	African-American (22%),	African-American (30%),	
	Asian (9%), Indian (13%)	Asian (13%), Indian (9%)	
	Other (4%)		
Nulligravid	11	12	0.768
Weight	147.1 (96–221) (6.733)	149.3 (100–235) (7.75)	0.987
Cycle day start	8.8 (2–14^a^) (0.613)	8.2 (3–12) (0.603)	0.514
Time to event	29.7 (21–42) (1.405)	30.61 (22–40) (1.283)	0.650
Days on therapy	33.4 (28–45) (1.152)	33.2 (23–43) (1.177)	0.916
Patients missing > 2 doses	0	1	0.312
Patients with breakthrough bleed	2^a^	10	**0.007**
Average day bleed occurred	16.5 (14–19)	15.25 (10–23) (1.47)	NA
Amount breakthrough bleeding	1	1.5 (1–3) (0.726)	0.321
Mean weight gain on therapy Of those, > 5 lbs. persistence at 1wk	3.6 (0–7) (0.469)	2.2 (0–6) (0.435)	**0.023**
	0	0	
Patients with side effects	5	12	0.054
Side effects	bloating (3), spotting (2)	bloating (2), spotting (9)	
	weight gain (4), nausea (1)	heavier bleeding (1), nausea (3)	
	headache (1)	headache (2), sore breasts (2)	
Days stop therapy to menses	3 (2–4) (0.167)	3.17 (2–4) (0.136)	0.537
Patients reporting light/normal flow	13	20	**0.047**
Reported duration of flow as normal or shorter	15	14	0.395
Satisfaction Score	4.09 (3–5) (0.153)	3.56 (2–5) (0.176)	**0.041**
Likely to use method again	4.13 (3–5) (0.158)	3.522 (2–5) (0.165)	**0.013**
Likely to recommend method	4.17 (3–5) (0.162)	3.39 (2–5) (0.137)	**0.002**
Unintended pregnancies	0	0	
Desire to conceive in 1 year	4	5	0.710
Time to conceive (months)	2.0 (1–3) (0.170)	3.6 (3–5) (0.186)	**0.032**

(^a^One patient in the treatment group was mistakenly enrolled at cycle day 14 and has been included in the analysis). Mean, range and standard error of the mean are shown

The bold on the rightmost column is for *p* <0.05 as significant

## Discussion

Most birth control methods have similar side-effects during the initiation of therapy, namely intermenstrual bleeding. They are designed more for long-term contraception rather than for episodic retiming of menses and each method has drawbacks and limitations.

Combined OCPs prevent pregnancy mainly through progestational effects. Menstrual flow is reduced as suppression of the HPAO axis produces a thinner and atrophic endometrium [7]. Starting treatment early in the cycle, especially in the early follicular phase (days 2–6), allows for early cycle capture. However, patients who desired postponing their menses were included in the study on the same day they presented to clinic provided they were on or before cycle day 12. Study and control groups were comparable as far as timing of treatment (day 8.8 ± 0.6 vs 8.2 ± 0.6), but within each group there was no statistical significance between bleeding and day treatment was initiated (not shown). While our study suggests that patients treated with norethindrone were significantly less likely to report subjectively lighter or normal periods when compared to controls, the clinical relevance is unclear. Further studies using objective measures, such as alkaline-hematin assays or a pictorial bleeding assessment, may be performed to objectively compare blood loss from norethindrone versus OCP withdrawal. While continuous regimens prevent menstruation by forgoing progesterone withdrawal, breakthrough spotting is typically encountered by 30–50% of women within 3 to 6 months of onset of therapy [8]. This was consistent with our findings where 2 (50%) women in the first study and 10 (43%) patients in the second study (both serving as controls), reported spotting in the first three months of use. “Low-dose” 35 μg ethinyl estradiol tablets have a slightly better spotting profile than 20 μg formulations but still do not eliminate spotting. For this study, the authors chose Ortho Cyclen because it is cheaply available as a generic drug (Sprintec, Teva) making it the most commonly prescribed OCP to uninsured patients in the United States and because its estrogen content could minimize breakthrough bleeding. Further investigations of breakthrough bleeding with OCPs containing other formulations of estrogens (such as estradiol) or progestins (dienogest, cyproterone acetate) are warranted as they have different half-life and receptor binding affinity as well as effect on sex hormone binding globulin. While OCPs are effective at reducing menstrual volume long-term, the high prevalence of vaginal spotting during initiation makes their use inappropriate in our study’s patient population. The spotting and side-effect profile may have contributed to lower satisfaction scores and willingness to try the method again or recommend this method.

Estrogen containing methods, such as combined OCPs (COC), are contraindicated in patients with hypercoagulable states, risk for thromboembolism, thrombophilia, inherited coagulation disorders, history of pulmonary embolus or autoimmune diseases [9, 10]. In addition, patients with a history of breast cancer, migraines with auras, liver cirrhosis, hypertension and smoking over age 35, are at increased cardiovascular risk when taking COCs [10] and should not take them. COCs are not recommended to postpartum patients within 30 days of delivery [11] due to the risks of VTE as well as a concerns regarding lactation [12].

NuvaRing (Organon) and Seasonique (Teva) are two alternative methods which have been studied for extended cycling and reduction of bleeding frequency. Seasonique (levonorgestrel/ethynyl estradiol) allows patients to menstruate four times yearly, however rates of breakthrough bleeding were highest at introduction (12 days) than in the third cycle (4 days) [13]. NuvaRing, a flexible transparent vaginally inserted device containing etonorgestrel and ethynyl estradiol [14]. This contraceptive is associated with breakthrough bleeding at initiation (3.5% of patients) and then again after the 16th day of continuous use with a peak incidence on day 29 [15]; the overall rate of spotting was 17.6% over the first 28 days [16]. Both methods contain estrogen and have the same limitations as described previously.

The levonorgestrel 20 mcg/day IUD is a LARC method that is FDA approved for the management of menorrhagia [17]. It functions as a contraceptive through its release of a progesterone agonist, thickening cervical mucus, expressing glycodelin A and thinning out the endometrial lining. IUDs also create an inflammatory environment in the uterine cavity [18]. By downregulating endometrial estrogen receptors, levonorgestrel induces endometrial thinning, glandular atrophy and stromal decidualization [18–20]. These devices, which last 3–5 years can produce hypomenorrhea or amenorrhea over the long-term. Comparing levonorgestrel IUD to OCPs, there was a greater patient satisfaction with IUDs when attempting to control menstrual bleeding [17]. However, when initially placed, IUDs are associated with spotting and increased bleeding [9]. This effect was a major determinant for discontinuation of IUD therapy [17]. In one prospective study, 25% of levonorgestrel users experienced spotting in the initial 6 months of therapy [21]. While this therapy establishes adequate long-term control of uterine bleeding, it is unsuitable for women seeking immediate control of bleeding and noninterference in fertility. Unlike COCs, levonorgestrel poses no cardiovascular, metabolic or thrombotic risks.

DMPA, an injectable progesterone analogue, suppresses ovulation [22], thickens cervical mucus and causes endometrial atrophy [23]. Adverse effects include enhanced fat deposition, decreased bone mineral density (resulting in an FDA black box warning) and weight gain [23]. DMPA has been shown to induce amenorrhea with long-term use. About 62% of women undergoing injections at appropriate intervals achieved amenorrhea within one year of use [24]. Short term, in a study of 722 women, all participants experienced spotting within 30 days of initial injection with 50% of women experiencing bleeding within the first week of therapy [24]. Thus, this treatment modality is unsuitable for women who desire postponing spotting in the short term. Furthermore, return to fertility may be delayed by DMPA. While 113 of 121 women studied returned to normal menstrual cycles within 6 months of discontinuation [25]*,* only 62.4% of 135 women who discontinued DMPA to conceive were able to achieve pregnancy by 6 months [26]. This method would be unsuitable for women who desire to conceive in the short term.

Similarly, Etonogestrel (Implanon), a highly effective LARC progestational implant designed for long-term contraception [27], is unsuitable for the short-term delay of menses. Implanon may cause irregular bleeding with 6.7% of patients experiencing frequent bleeding and 17.7% complaining of prolonged bleeding while spotting occurred unpredictably [28]. Return to fertility may be delayed: in a study of 80 women who discontinued Implanon for desired pregnancy, only 48.8% became pregnant within one year [29]*.*

Norethindrone is used to treat abnormal uterine bleeding, amenorrhea, endometriosis, and contraception [30]. It blocks the anterior pituitary gland from releasing LH (inhibiting ovulation) and downregulates estrogen receptors on the endometrium, limiting proliferation. It also enhances glandular secretion and prevents breakdown of the endometrium until its withdrawal [9, 19, 31]. Continuous administration of a progesterone analog induces amenorrhea. For this reason, norethindrone has anecdotally been used for years in Jewish circles [6]. One publication reported no episodes of vaginal bleeding in a limited study where patients were prescribed norethindrone to delay menses [6]. This is consistent with our results and reinforces the effectiveness of norethindrone when attempting to immediately delay menstruation.

Weight gain, a common side-effect [32], may lead to poor compliance and breakthrough bleeding. Additional adverse effects include nausea, headache, acne, breast tenderness and bloating [33]. Nine women (33%) complained of bloating. The average weight gain with norethindrone (3.6 pounds) may be attributable to hormonally induced water retention as its onset and resolution was rapid and coincided with the initiation and discontinuation of treatment. A mild diuretic may be used to alleviate this side-effect during treatment. Norethindrone’s relatively short half-life, about 9 h, requires careful compliance with dosing regimens [34]. If a patient misses doses, she is at risk of withdrawal bleeding and, potentially, ovulation. None of the study patients recalled missing > 2 doses. When compared to OCPs, norethindrone has minimal pro-coagulant effects, making it an excellent choice in patients at risk for VTE or cardiovascular disease [35].

Another advantage of norethindrone for women wishing to delay vaginal bleeding without intending for long-term contraception is the rapid return to fertility. Compared to other hormonal formulations, norethindrone weakly suppresses the hypothalamic-pituitary-ovarian axis allowing for a quicker return to fertility after discontinuation [36] and this is supported by our findings. It is unclear, however, if this difference in time to conceive (2 versus 3.6 months in treatment and controls respectively) is of clinical relevance. Typically, withdrawal bleeding occurs within 24–72 h [37] as confirmed in our investigation.

## Conclusion

Many women present to their OBGYN requesting to retime menstruation. While practitioners typically start these patients on OCP’s, norethindrone may be a superior alternative. Furthermore, when there is insufficient time for the cycle to regulate itself or when any amount of spotting cannot be tolerated, traditional methods such as OCPs and IUDs are inadequate. In this pilot study, norethindrone demonstrated its ability to delay menses without causing spotting and was well tolerated despite causing mild weight gain. Unlike contraceptives, norethindrone allows for an immediate return to fertility after discontinuation. Alternatively, a transition to LARCs or hormonal methods can be initiated, if desired, with reasonable assurance that pregnancy is excluded.

Limitations in this study include the small number of participants, the enrollment of patients on or before cycle day 12, comparison to only one formulation of OCPs, the use of identifiable tablets and the lack of objective measurement of blood loss during withdrawal bleeding. Further studies may use other formulations of OCPs, particularly based on norethindrone and other drug delivery methods, such as vaginal rings. It would also be interesting to determine if breakthrough bleeding occurs at higher rates when treatment is initiated later in the cycle. Further work will consist of an extended randomized double-blinded study with greater power where variations in dosage regimens may be explored.

## Abbreviations

BMI: Body Mass Index
COC: Combined Oral Contraceptive Pills
DMPA: Depot-Medroxyprogesterone Acetate
FDA: Food and Drug Administration
HPAO: Hypothalamic-Pituitary-Adrenal-Ovarian Axis
IRB: Institutional Review Board
IUD: Intrauterine Device
LARC: Long Acting Reversible Contraceptive
LH: Luteinizing Hormone
mg: milligram
NIH: National Institute of Health
OBGYN: Obstetrician-Gynecologist
OCP: Oral Contraceptive Pill
TID: Three Times Daily
VTE: Venous Thromboembolism
